# What roles matter? An explorative study on bullying and cyberbullying by using the eye‐tracker

**DOI:** 10.1111/bjep.12604

**Published:** 2023-04-26

**Authors:** Laura Menabò, Grace Skrzypiec, Phillip Slee, Annalisa Guarini

**Affiliations:** ^1^ Department of Psychology “Renzo Canestrari” University of Bologna Bologna Italy; ^2^ Department of Education Flinders University Adelaide South Australia Australia

**Keywords:** bullying, cyberbullying, eye‐tracking, roles, school

## Abstract

**Background:**

Bullying and cyberbullying are serious public health concerns that involve more roles beyond the bully and the victim (pro‐bullies, defenders, bystanders). However, students often perceive the phenomena as dyadic.

**Aim:**

The purpose was to examine students' perceptions of different roles when observing bullying and cyberbullying scenes combining implicit (attention by using the eye‐tracker) and explicit (verbal reports) measures.

**Sample:**

We included 50 Italian students (aged 10–11).

**Methods:**

Students watched 12 drawings of different types of bullying and cyberbullying while their gaze was tracked, and subsequently described each drawing verbally. We ran repeated measure ANOVAs to compare attentional indexes (fixation count, visit count and total fixation duration) in observing roles and Cochran's Q test to evaluate differences in the verbal identification of roles.

**Results:**

Overall, the victim and bully were the most observed and identified roles in every type of bullying and cyberbullying scenario. Concerning the other roles, a discrepancy was observed between the implicit and explicit measures since although it was greatly identified, the pro‐bully received less attention, and while the bystander received great attention, it was mentioned less. Finally, the defender was more observed and identified in physical bullying and cyberbullying.

**Conclusions:**

Our study points out for the first time the dyadic perception of the phenomena among adolescents using implicit and explicit measures and sheds light on differences among the roles in different forms of bullying. Further research including the eye‐tracker would be worthwhile given the possibility of exploring the phenomena from different perspectives.

## INTRODUCTION

Bullying is usually defined as a repeated act of intentional aggressiveness perpetrated by one or more students towards another student who cannot easily defend themselves and it can occur in different forms (Smith et al., 2013). Direct bullying is defined as targeting the victim explicitly, including physical aggression, such as hitting, kicking or pushing the victim, and verbal aggression, such as insulting or name‐calling. Indirect bullying is more covert and less explicit, including isolating the victim or spreading rumours (Rivers & Smith, 1994). Cyberbullying shares most of the characteristics of traditional bullying but includes some unique and idiosyncratic features that can result in widespread and continuing humiliation for the victim (Campbell & Bauman, 2018; Menesini et al., 2021; Tokunaga, 2010). Despite these differences, bullying and cyberbullying have shown significant overlap (Burton et al., 2013; Hase et al., 2015; Kowalski & Limber, 2013; Olweus, 2012). Both forms can adversely affect the well‐being of adolescents on multiple levels, being associated with internalizing and externalizing symptoms, as shown by meta‐analyses and reviews (see Guzman‐Holst & Bowes, 2021; Sigurdson et al., 2021). Stapinski et al. (2015), for example, found that adolescents who had been bullied were likely to exhibit anxiety and depression symptoms controlling for family, emotional and behavioural factors. Similarly, longitudinal studies have confirmed a strong association between cyberbullying, adolescents' depression (e.g. Hemphill et al., 2015) and low life satisfaction (e.g. Sumter et al., 2012). In addition, bullying and cyberbullying are associated with a significantly higher risk of suicide than those not involved (Hinduja & Patchin, 2010; Klomek et al., 2010; Koyanagi et al., 2019). For example, Kowalski et al. (2014) explored the role of cyberbullying on suicidal thoughts by conducting a meta‐analysis of 131 studies. They found that elevated stress levels and suicidal thoughts had the greatest effect sizes as associated with cybervictimization. While there is a significant history of research into bullying and cyberbullying and the associated impact on student mental health, the phenomena continue to represent a priority for researchers, policymakers and schools worldwide (Arseneault, 2018; Casper, 2021).

### Current approaches to bullying and cyberbullying

Over the past decade, data on bullying and cyberbullying have been collected using quantitative and qualitative methods (Dennehy et al., 2020; Maran & Begotti, 2021; Smith et al., 2021). Quantitative research, which includes self‐report measures by students and peer nomination by teachers and classmates, represents the most widely used methodology (Espelage & Swearer, 2003; Maran & Begotti, 2021). The strength associated with self‐report approaches is the validity and reliability of the tools that provide essential information about the individual's perceptions of the frequency and intensity of peer aggression (Hunter et al., 2021; Maran & Begotti, 2021). However, self‐reported questionnaires exhibit limitations, as students could under‐report their involvement or they may answer in a socially desirable manner (Berne et al., 2013; Rigby & Johnson, 2006). Furthermore, the lack of common definitions across different questionnaires raises doubts about the validity of findings in measuring bullying (Cornell & Bandyopandhyay, 2010; Hunter et al., 2021; Volk et al., 2017). Alternatively, approaches assessing bullying status based on peer and teacher nomination provide independent information on victims, type of aggression and student involvement (Espelage & Swearer, 2003; Pellegrini & Bartini, 2000). However, an important source of concern includes possible prejudice or relationship problems not related to bullying (Hymel et al., 1990; Volk et al., 2017).

Concerning qualitative research, studies conducted through interviews or focus groups have enabled exploration, detailed descriptions and interpretation of individual experiences and young people's perceptions of bullying and cyberbullying (Creswell, 2013; Hutson et al., 2018; Maran & Begotti, 2021; Mishna et al., 2008). However, methodological issues arise, such as the methods of data analysis that may be too flexible or not well formulated (Silverman, 2013). Likewise, qualitative researchers often emphasize the importance of data over theory (Corbetta, 2003). Therefore, overall, methods used to understand the phenomenon of bullying are subject to limitations.

### A new approach: Eye‐tracking

Given the limitations of the current research approaches, more techniques that allow for a different comprehension of the phenomena may be meaningful in shedding further light on bullying and cyberbullying. One yet relatively untapped approach is to use eye‐tracking, which records the movements of people's eyes while interacting with visual stimuli such as images, text, websites and video clips. Eye‐tracking is based on the ‘eye‐mind’ hypothesis proposed by Just and Carpenter (1976), which asserts that eye movements provide dynamic feedback regarding where attention is being directed, and it is, therefore, considered a validated method of studying visual attention with millisecond precision (Vraga et al., 2016). Using near‐infrared light, eye tracking captures the eye's exact position and gaze point on a screen, providing information on visual attention. Over the years, it has been proposed that visual attention is divided into two categories: top‐down and bottom‐up processing (Buschman & Miller, 2007; Mancas, 2009). The bottom‐up is often thought of as ‘stimulus‐driven’, where the attention is instantly drawn to the most salient stimuli or feature that evokes stronger neural activation (Desimone & Duncan, 1995; Wolfe, 1994). Using this type of process, we can select the most relevant stimuli while filtering out the irrelevant ones, allowing us to respond quickly to different situations (Katsuki & Constantinidis, 2014). By contrast, the top‐down process happens when the information is actively sought out in the environment, and it is usually guided by motivation, a priori knowledge and goals (Connor et al., 2004; Corbetta & Shulman, 2002; Itti & Koch, 2001; Vraga et al., 2016). Moreover, because attentional control processes represent implicit measures, largely unconscious and based on habits, participants have difficulty adjusting their behaviour based on expectations from the experimenter or social prestige (Frey et al., 2000; Graham et al., 2012; Oar et al., 2022; Vraga et al., 2016; Weber et al., 2015). Over the years, eye‐tracking technology has been widely applied to the study of cognitive processes and emotional responses such as spatial attention and competitiveness in social decision‐making (Giacomantonio et al., 2018; Guarini et al., 2021; Koornneef & Vanberkum, 2006). However, few studies have used it to investigate bullying and cyberbullying.

### Bullying, cyberbullying and eye‐tracking

Limited research has used eye‐tracking to examine bullying and cyberbullying albeit with different aims. Caravita et al. (2016) investigated if bullying and cyberbullying interactions may attract more attention than prosocial and neutral interactions by using eye‐tracking in a sample of young adults. They found that bullying and cyberbullying videos captured more attention overall, while the prosocial video had the lowest number of observations. Interestingly, they found that those who were victimized, assessed through the use of self‐report questionnaires, diverted their early attention away from bullying and cyberbullying videos, to avoid negative emotional reactions already experienced in the past. In a different study, Troop‐Gordon et al. (2019) found that preadolescents' high attention to the bully in video clips was associated with high levels of aggressiveness in victims as measured by questionnaires. By contrast, attention to the victim was negatively correlated with aggression tendency regardless of the level of victimization. In a following study, McConnell and Troop‐Gordon (2021) showed that high attention to the bully in severely victimized early adolescents was associated with less seeking assistant. Indeed, they found that severely bullied victims tended to pay less attention to the adults in the video clips, who potentially could have helped to stop the aggression. In the authors' opinion, focusing on bullies may reinforce victims' perceptions of a hostile peer environment, leading to a sense of hopelessness that may, in turn, result in a lack of attention to adults who may be able to help (McConnell & Troop‐Gordon, 2021). In light of these studies and considering the benefit of using eye‐tracking, this technology can investigate students' attention to bullying and cyberbullying phenomena, potentially overcoming the various limits of quantitative and qualitative research methods (Volk et al., 2017). The attention assessed by the eye‐tracker, however, represents an implicit measure. To provide a better picture of the investigated phenomena, therefore, it has been suggested to combine implicit (e.g. attention) and explicit measures (e.g. interviews) (DeCoster et al., 2006; Giesbrecht, 2019).

### Bullying involvement roles

There is broad agreement today that bullying and cyberbullying are social phenomena occurring in many different settings, including school classrooms and online environments (Cross et al., 2015; Gini et al., 2021; Salmivalli et al., 1996; Zych et al., 2015; Zych et al., 2018). From a theoretical perspective, the nature of these dynamics, why they occur and what motivates them, can be interpreted in the light of Social Information Processing theory (Crick & Dodge, 1994). The theory states that students interpret peer interactions based on information that includes encoding and interpreting social cues, clarifying goals, developing strategies for achieving those goals, evaluating strategies and responding (see Crick & Dodge, 1994). Considering the complexity of interpreting peer interactions and in particular aggressive peer interactions, such as bullying and cyberbullying, the first step is thus to understand and recognize the different roles of individuals involved in bullying and cyberbullying using an analysis of social cues and responses. Three common roles beyond the dyad of bully‐victim have been identified: students who side with the perpetrator (assisting and reinforcing behaviour), aid the victim (upstanding and defending behaviour) or ignore the event (passive behaviour; Salmivalli et al., 1996; Salmivalli et al., 2011). Regarding individuals who side with the bully, two different roles have been highlighted: bully ‘assistants’, who do not start the bullying but join in the aggression, and the bully ‘reinforcers’, who encourage bullying by laughing, watching and inciting the bully (Salmivalli et al., 1996; Salmivalli, 2010). While there are conceptual differences between the reinforcer and assistant, the characteristics and association of these two roles, as well as the factor structure of the instruments used, indicate that they are nearly impossible to distinguish empirically (Gini et al., 2021). Instead, it has been suggested that they may be combined to form one category, labelled ‘pro‐bully’ (Jungert et al., 2016; Nocentini et al., 2013; Thornberg & Jungert, 2013). Countering the bully, one role that has been identified in support of the victim is that of a ‘defender’. Defenders are peers who act in episodes of bullying by standing up for victims, reporting the incidents to significant adults or comforting victims (Xie & Ngai, 2020). Separate from these roles are those pupils who withdraw from the scene, deny that bullying is taking place, become avoidant onlookers or remain silent. They can be categorized as ‘passive bystanders’ (Gini et al., 2008; Cowie, 2000; Menesini et al., 2003; Pozzoli & Gini, 2010; Thornberg et al., 2017).

Similar to traditional bullying, roles have been identified in cyberbullying (Menesini & Nocentini, 2009). The pro‐bully can support cyberbully's hurtful actions by sharing, forwarding or reinforcing the message, the defender plays an active role in comforting the victim or reporting incidents to adults, while the passive bystander remains an inert observer, refusing to intervene to help the victim (Menesini et al., 2003; Pozzoli & Gini, 2010; Salmivalli et al., 2011).

However, despite these bullying involvement roles, it is important to note that bullying and cyberbullying are often considered dyadic phenomena by students, where the focus is just on the bully's and victim's characteristics. Indeed, previous research has highlighted that students are not always aware of the different bullying involvement roles (e.g. Slonje et al., 2013), as they consider bullying and cyberbullying as phenomena involving just the bully and the victim (Bosacki et al., 2006; Guarini et al., 2019; Mameli et al., 2022; Thornberg & Knutsen, 2011).

A qualitative study by Bosacki et al. (2006) using arts‐based methods revealed that students between the ages of 8 and 12, depicted the bully‐victim dyad in 93% of their drawings, while only 7% portrayed a more complex group process. A dyadic perception also emerged in a qualitative analysis of students' explanations, which indicated that a large majority attributed bullying just to the individual, while a small minority attributed it to the peer group (Thornberg & Knutsen, 2011). Similar results have been reported concerning cyberbullying, where the roles of the cyberbully and the cybervictim appeared in a greater number of vignettes of comics produced by adolescents (Mameli et al., 2022). Likewise, Guarini et al. (2019) found that just around 1% of students aged 11–14 cited roles other than the bully and victim when asked to describe who was involved in the cyberbullying dynamics.

Studying the awareness of the different roles involved in bullying and cyberbullying could be essential to implementing effective interventions. Awareness that other students are involved in bullying and cyberbullying dyamics may represent the first step to promoting student responsibility and changing the normative rules that may also support and foster aggressive behaviour both face‐to‐face and online (Haataja et al., 2015). Therefore, it is important to gain more insight into the perception of different roles involved in bullying and cyberbullying.

### Aims

The purpose of this study was to understand the differences among the roles, namely bully, victim, pro‐bully, defender and bystander, in attracting the attention of students watching bullying and cyberbullying scenarios. In line with previous research on bullying and cyberbullying, it was expected that participants would pay more attention to the bully and victim, suggesting a dyadic conception of the phenomena.

We sought to use eye‐tracking to examine differences among the roles using three attentional indexes:

- Fixation counts (number of times the participant fixates on a role);
- Visit counts (number of visits ‐step in and out‐ of a role);
- Total fixation durations (total duration for all fixations in a role).

In light of the research findings discussed above, we hypothesized that the bully and victim would receive a greater number of fixation counts, indicating a need for students to explore with more attention the different parts of the bully and the victim's characters than the other roles. Furthermore, we hypothesized that the bully and the victim would also receive more visits, meaning that students would have a greater need to step in and out more often in these roles to observe them than in the other roles. Finally, we predicted that the fixation duration would vary with the bully and victim roles watched for longer than the other roles, confirming their relevance in the scene.

Furthermore, to analyse the perceived salience of roles and their identification by students, we also examined the description of vignettes using students' own words as we asked them to describe each vignette after the eye‐tracking task. In this case, we hypothesized a greater verbal identification of the bully and the victim compared with the other roles. In addition, we also hypothesized differences between eye‐tracking results and interviews, in line with previous studies on other topics (Giesbrecht, 2019). Indeed, on the one hand, the eye‐tracker provides a measure of implicit behaviour, such as the attention displayed, which cannot be detected by introspection or self‐report. Interviews, on the other hand, allow the investigation of explicit processes, such as conscious thought described by the individual during the process (DeCoster et al., 2006). Thus, using implicit and explicit measures can help improve our understanding of student awareness of the different roles involved in bullying and cyberbullying.

## METHOD

### Participants

We contacted through e‐mail the principal of one lower secondary public school in Bologna (Emilia‐Romagna region) in October 2021 and received a positive response. Thus, data were collected from three first‐year (grade 5) classes. Eleven students from different classes were not present at school during the days of data collection, so they were not included in the sample. A final sample of 50 students volunteered to participate in the study and watched bullying scenarios in a room using eye‐tracker technology at their school. Students were aged 10–11 years (*M* = 10.3 years, *SD* = 3.4 months). All participants lived in Bologna, in the North of Italy. They were Italian except for one student who was born in Saudi Arabia. Concerning the gender composition, 27 students (54%) were males and 23 (46%) were females. The data were collected from December 2021 to January 2022.

### Ethics

The study protocol met the ethical guidelines for the protection of human participants, including adherence to the legal requirements of Italy, and received formal approval by the Bioethics Committee of the first and last authors' University. The parents of the children provided their informed written consent for participation in the study, data analysis and anonymous data publication. No economic incentives were provided to parents or students.

### Stimuli and apparatus

#### Stimuli

**FIGURE 1:**
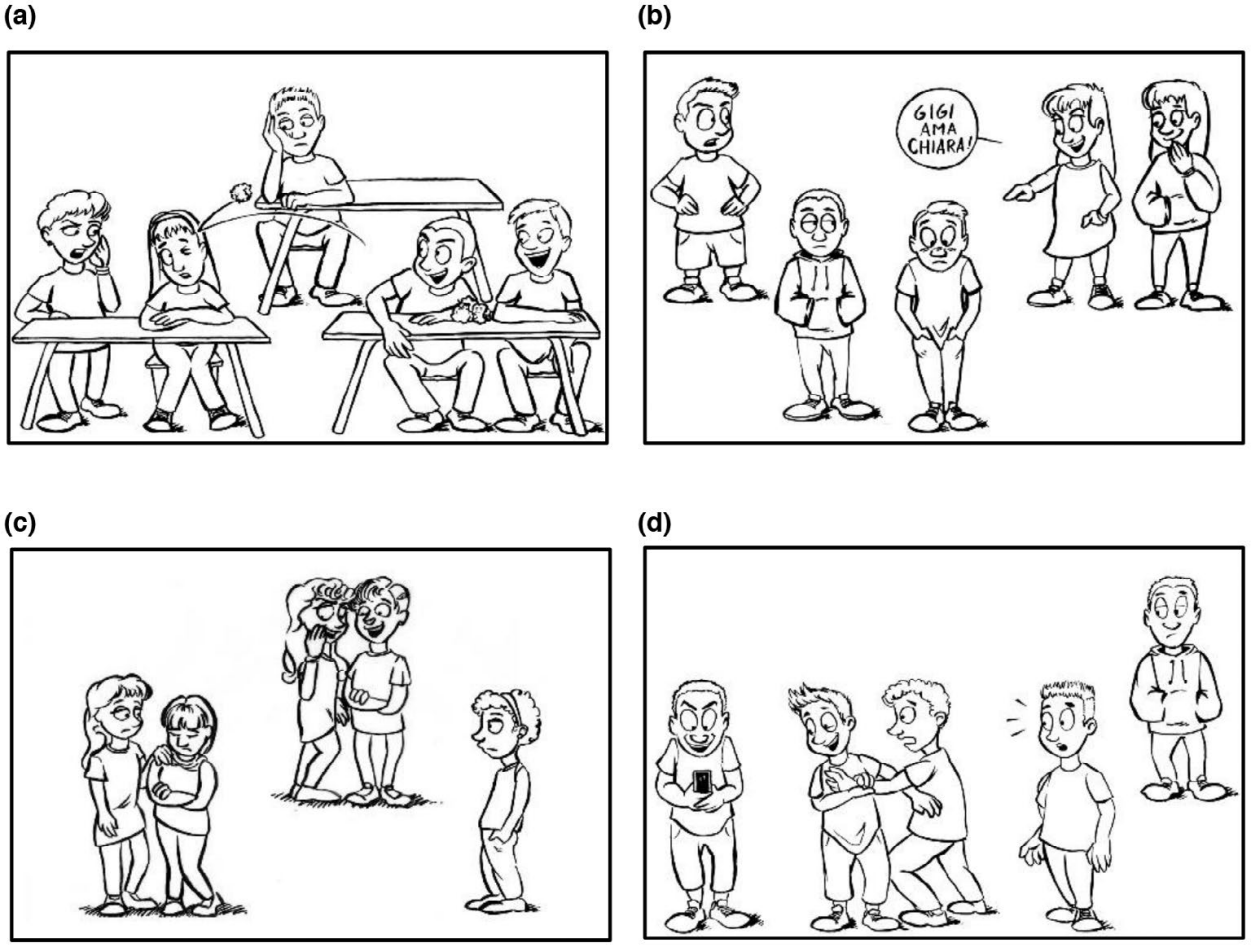
Examples of the vignettes. The figure shows four of the 12 vignettes that students watched during the experiment. 1 (a) physical bullying, 1 (b) verbal bullying, 1 (c) relational bullying and 1 (d) cyberbullying. The verbal bullying phrase means ‘Gigi loves Chiara’.

Stimuli for the eye‐tracking task were composed of 12 vignette drawings representing different types of bullying episodes (Figure 1). There were three drawings for physical bullying, three for verbal bullying, three for relational bullying and three for cyberbullying. All roles (bully, victim, pro‐bully, defender and bystander) were represented in each scene. The vignettes were drawn by a professional artist specifically recruited for the present study and were based on guidelines derived from existing research. Indeed, the selection of the different types of bullying scenes represented in each vignette was based on the representativeness of bullying experiences for young people, as suggested by several authors (Aricak et al., 2008; Patchin & Hinduja, 2006; Seals & Young, 2003). The creation of the stimuli was also based on Troop‐Gordon et al. (2019), in which six scenes of physical bullying, six of verbal bullying and six of relational bullying were developed. For each type of bullying (physical, verbal and relational), we chose three out of the six acts described by Troop‐Gordon et al. (2019). Three other drawings illustrated acts of cyberbullying and one of them was based on the video by Caravita et al. (2016).

The vignettes were gender‐balanced. For each type of bullying, one vignette represented only males, one only female, and one was mixed‐gender. In summary, the following acts of bullying are depicted.

- Physical bullying: pushing (males), tripping (females), throwing paper balls (mixed, see Figure 1a). In the mixed‐gender vignette, the bully, the pro‐bully and the bystander were males, while the victim and defender were females.
- Verbal bullying: forcing someone to do someone else's homework (males), teasing about someone's body odour (females), mocking a student's attraction to another student (mixed, see Figure 1b). In the mixed‐gender vignette, the bully and pro‐bully were females, while the victim, defender and bystander were males.
- Relational bullying: excluding someone from a game (males), spreading rumours (females, see Figure 1c), refusing offered food (mixed). In the mixed‐gender vignette, the bully, pro‐bully and defender were males, while the victim and bystander were females.
- Cyberbullying: stealing another person's phone (males, see Figure 1d), making fun of someone else when watching the phone (females), taking a photo of another person (mixed). In the mixed‐gender vignette, the bully, defender and bystander were females, while the pro‐bully and victim were males.

Each drawing was black and white to ensure the context was as neutral as possible and that participants would not be attracted to other stimuli present in the scene not directly related to the bullying episode (such as colours).

#### Apparatus

The Tobii Pro X2/60 recorded participants' eye movements, sampling gaze location at 60 Hz. The different role in each scene was considered a separate area of interest (AOI) for this study. The AOI is a chosen portion of selected regions in a stimulus that allows extracting metrics specific to those locations. Therefore, each vignette presented five different AOI, one for each of the different roles (i.e. bully, victim, pro‐bully, defender, bystander) presented in the scene.

The drawings were displayed to children on a 19‐inch monitor at 1600 × 900 pixels resolution. The recommended configuration for children and pre‐adolescents, which follows a 5‐point calibration procedure, was used (Dys, 2019). The calibration process ensured that the eye‐tracker memorized the characteristics of the participants' eyes and calculated the direction of their gaze on the surface of the screen.

### Procedure

The first author, with the help of an expert psychologist in bullying and cyberbullying, conducted the experiment in a dedicated room at the students' schools. After students were seated at a table and in front of the screen, they were told their eye location would be recorded as they watched vignettes about bullying and cyberbullying. The eye‐tracker was then calibrated and validated to ensure a gaze position accuracy of .50 degrees or better. Students could decide when they wanted to move to the next image by pressing the right arrow on the keyboard. After each student had completed the experiment, the vignettes were presented again on the computer screen but without recording eye movements. Students were invited to describe in a few words what happened in each vignette. The researcher avoided mentioning the different roles involved or posing questions in order not to induce or manipulate responses. As each student was interviewed, the researcher faithfully reported word‐for‐word descriptions of all the pictures.

The experiment took approximately 10 minutes to complete.

### Coding

Concerning the eye movements, the following metrics were recorded from each AOI: fixation counts (number of times the participant fixates an AOI), visit counts (number of visits to an AOI) and total fixation durations (in milliseconds, total duration for all fixations within an AOI). The metrics were extracted using Tobii Studio software.

Interview data were analysed for content by considering, in the first instance, the presence or absence of each role and then if the role was mentioned, whether the description attributed to them was consistent with the role's function. In each vignette, a score of 0 was assigned if the role was absent, 1 if the role was present and understood correctly and 2 if the role was present but the action was inconsistent with the character's role.

We report three examples of the description of vignettes to which we assigned a score of 0, 1 and 2.

Example 1: ‘*There is a girl who is saying "Gigi loves Chiara", so she is embarrassing him*’ (vignette 1b, see Figure 1, English translation). In this example, the bully and the victim are correctly mentioned and identified (score= 1) while the other roles are not mentioned (score= 0).

Example 2: *‘There is one child next to another one and he throws balls at her, the other next to him laughs, the other says stop, and the other behind observes the scene’*(vignette 1a, see Figure 1. English translation). In this example, all the roles are correctly mentioned and identified (score= 1).

Example 3: ‘*One boy took the phone from another. The friend of the one who took it holds the other so that he does not take it. Two boys watch without interacting*’ (vignette 1d, see Figure 1. English translation). The bully, the victim, the pro‐bully and the bystander were correctly identified (score= 1) while the defender was not recognized in its role, but rather it was mistaken for another bystander (score= 2).

Consequently, each role within the different types of bullying was coded 150 times (50 participants describing three vignettes representing each type of bullying and cyberbullying). The first author analysed and coded all the interviews, while the last author analysed and coded 20% of the randomly assigned interviews. Intercoder reliability (Cohen's kappa) was performed and ranged from .86 to 1.

### Data analyses

To achieve our aim, we extrapolated the attentional indexes (fixation counts, visit counts and total fixation durations) for each role (bully, victim, pro‐bully, defender, bystander) from each vignette. We computed the means of the attentional indexes of each role for each type of bullying (physical, verbal and relational) and cyberbullying.

We ran repeated measure ANOVAs, using SPSS v28, considering partial eta squared (*η*_*p*_^*2*^) effect sizes to understand the differences in fixation counts, visit counts and total fixation durations among roles within each type of bullying. Four repeated measures, ANOVAs, one for each type of bullying (physical, verbal, relational and cyberbullying), were undertaken with fixation counts as the dependent variable and the different roles as the independent variable (bully, victim, pro‐bully, defender and bystander). The same procedure was followed for analysing differences in visit counts and total fixation durations as the dependent variables. When a significant difference was found, post hoc tests, with Bonferroni adjustment, considering Cohen's *d* effect size, were performed.

Regarding the verbal description, once the coding was complete, we analysed interview data and assigned a value of 1 to responses where the different roles were correctly mentioned and understood (value 1) in each bullying scenario. Then we used Cochran's Q test, a non‐parametric test to analyse this numeric data. Cochran's Q test operates in the same way as a one‐way repeated‐measures ANOVA; however, it is applied to non‐continuous data to determine whether the proportion of ‘successes’ (value = 1 in our case) is the same across groups. When significant differences were found, pairwise comparisons were run.

## RESULTS

### Fixation counts

A significant role effect on the fixation counts in each type of bullying was found (Table 1). In all scenarios, there was a greater fixation on the victim and bully than on any other roles.

**TABLE 1:** Means, standard deviations and one‐way analyses of variance in the number of fixation counts among roles in different forms of bullying and cyberbullying.

	Bully (A)		Victim (B)		Pro‐bully (C)		Defender (D)		Bystander (E)		*F*(1,49)	Bonferroni post‐hoc
	*M*	*SD*	*M*	*SD*	*M*	*SD*	*M*	*SD*	*M*	*SD*		
Physical bullying	7.97	4.17	9.60	5.16	3.12	3.29	5.78	3.81	4.68	3.54	56.63***	B > A A,B > C,D,E D,E > C
Verbal bullying	6.17	3.89	6.93	4.19	2.96	2.18	3.48	2.25	4.51	2.87	27.75***	A,B > C,D,E E > C
Relationalbullying	7.00	4.46	6.86	3.96	3.82	3.24	3.51	2.74	4.54	2.38	28.09***	A,B > C,D,E
Cyberbullying	8.59	4.44	9.34	5.18	5.63	3.79	6.79	3.75	4.77	2.97	29.65***	A,B > C,D,E D > E

*** p < .001.

Concerning physical bullying, *F*(1, 49) = 56.63, *η*_*p*_^*2*^ = .54, *p* < .001 (Table 1), there was a greater number of fixations on the victim (*M* = 9.60) with a medium effect size compared with the bully (*M* = 7.97, *p* = .002, Cohen's *d* = .58) and large effect sizes compared with the other roles (defender: *M* = 5.78, *p* < .001, Cohen's *d* = 1.26; bystander: *M* = 4.68, *p* < .001, Cohen's *d* = 1.13; pro‐bully: *M* = 3.12, *p* < .001, Cohen's *d* = 1.53). Fixations on the bully were greater compared with the defender (*p* < .001, Cohen's *d* = .72), the bystander (*p* < .001, Cohen's *d* = .84) and the pro‐bully (*p* < .001, Cohen's *d* = 1.40), while fixations on the bystander and the defender were greater than the mean fixation on the pro‐bully (*p =* .004 and *p* < .001, Cohen's *d* = .54 and .95).

A significant role effect was also found in verbal bullying, *F*(1,49) = 27.75, *η*_*p*_^*2*^ = .40, *p* < .001 (Table 1). A higher number of fixations on the victim (*M* = 6.93) and the bully (*M* = 6.17) with large effect sizes compared with other roles (bystander: *M* = 4.51, *p* < .001 and *p* = .041, Cohen's *d* = .74 and .43; defender: *M* = 3.48, *p* < .001 for both the comparisons and Cohen's *d* = .98 and .75; pro‐bully: *M* = 2.96, *p* < .001 for both the comparisons and Cohen's *d* = 1.09 and .86) were observed. Finally, there was a greater number of fixations on the bystander compared with the pro‐bully (*p* < .001, Cohen's *d* = .61).

Relational bullying also presented a significant role effect, *F*(1,49) = 28.09, *η*_*p*_^*2*^ = .36, *p* < .001 (Table 1). Bonferroni post‐hoc revealed that the bully (*M* = 7.00) and the victim (*M* = 6.86) presented higher fixation counts with large effect sizes compared with all the other roles (bystander: *M* = 4.54, *p* < .001 for both the comparisons, Cohen's *d* = .61 and .69; pro‐bully: *M* = 3.82, *p* < .001 for both the comparisons, Cohen's *d* = 1.00 and 1.09; defender: *M* = 3.51, *p* < .001 for both the comparisons, Cohen's *d* = .99 and 1.21).

A significant role effect was confirmed in cyberbullying, *F*(1,49) = 29,65, *η*_*p*_^*2*^ = .38, *p* < .001 (Table 1). Bonferroni post‐hoc showed that the victim (*M* = 9.34) and the bully (*M* = 8.59) were the roles with the greatest number of fixation counts and large effect sizes compared with the othes roles (defender: *M* = 6.79, *p* < .001, Cohen's *d* = .88 for the victim; *p* = .012 and Cohen's *d* = .49 for the bully; pro‐bully: *M* = 5.63, *p* < .001 for both the comparisons, Cohen's *d* = 1.01 and .80; bystander: *M* = 4.77, *p* < .001 for both the comparisons, Cohen's *d* = 1.08 and 1.05). Moreover, the defender significantly presented a greater number of fixations compared with the bystander (*p* = .002, Cohen's *d* = .58).

### Visit counts

The second attentional index included was the visit counts (Table 2). Physical bullying showed a significant role effect, *F*(1,49) = 47.30, *η*_*p*_^*2*^ = .25, *p* < .001 (Table 2). Bonferroni's post‐hoc comparisons showed that the victim (*M* = 4.30) and the bully (*M* = 3.84) presented a significantly greater number of visit counts with large effect sizes compared with all the other roles (defender: *M* = 2.55, *p* < .001 for both the comparisons, Cohen's *d* = 1.50 and .94; bystander: *M* = 2.26, *p* < .001 for both the comparisons, Cohen's *d* = 1.08 and .85; pro‐bully: *M* = 1.73, *p* < .001 for both the comparisons Cohen's *d* = 1.25 and 1.35). The defender and the bystander also showed a significant difference compared with the pro‐bully (*p* = .011 and *p* = .047, Cohen's *d* = .49 and .42).

**TABLE 2:** Means, standard deviations and one‐way analyses of variance in the number of visit counts among roles in different forms of bullying and cyberbullying.

	Bully (A)		Victim (B)		Pro‐bully (C)		Defender (D)		Bystander (E)		F (1,49)	Bonferroni post‐hoc
	*M*	*SD*	*M*	*SD*	*M*	*SD*	*M*	*SD*	*M*	*SD*		
Physical bullying	3.84	1.83	4.30	2.02	1.73	1.58	2.55	1.39	2.26	1.40	47.30***	A,B > C,D,E D,E > C
Verbal bullying	3.85	1.69	3.75	1.83	1.79	1.21	1.69	.95	2.18	1.23	57.80***	A,B > C,D,E E > D
Relational bullying	3.74	2.04	3.69	1.99	2.35	1.82	1.99	1.34	1.75	.91	41.98***	A,B > C,D,E
Cyberbullying	3.10	1.63	4.19	2.16	3.07	2.01	3.49	1.69	1.81	.97	36.96***	B > A,C,D,E A,C,D > E

*** p < .001.

A significant role effect was also found in verbal bullying, *F*(1,49) = 57.80, *η*_*p*_^*2*^ = .30, *p* < .001 (Table 2). Bonferroni post‐hoc revealed that the bully (*M* = 3.85) and the victim (*M* = 3.75) presented a greater number of visit counts with large effect sizes than all the other roles (bystander: *M* = 2.18, *p* < .001 for both the comparisons, Cohen's *d* = 1.12 and 1.24; pro‐bully: *M* = 1.79, *p* < .001 for both the comparisons, Cohen's *d* = 1.33 and 1.21; defender: *M* = 1.69, *p* < .001 for both the comparisons, Cohen's *d* = 1.34 and 1.24). Moreover, the bystander presented a higher number of visit counts than the defender (*p* = .047, Cohen's *d* = .42).

A significant role effect was confirmed in relational bullying, *F*(1,49) = 41.98, *η*_*p*_^*2*^ = .22, *p* < .001 (Table 2). Post‐hoc comparisons showed that the bully (*M* = 3.74) and the victim (*M* = 3.69) received a greater number of visit counts with large effect sizes compared with all the other roles (pro‐bully: *M* = 2.35, *p* < .001 for both the comparisons, Cohen's *d* = 1.01 and .98; defender: *M* = 1.99, *p* < .001 for both the comparisons, Cohen's *d* = 1.10 and 1.23; bystander: *M* = 1.75, *p* < .001 for both the comparisons, Cohen's *d* = 1.15 and 1.11).

In relation to cyberbullying, ANOVA confirmed a significant role effect, *F*(1,49) = 36.96, *η*_*p*_^*2*^ = .27, *p* < .001 (Table 2). Bonferroni post‐hoc comparisons showed that the victim (*M* = 4.19) presented the greatest number of visit counts with large effect sizes compared with the other roles (defender: *M* = 3.49, *p* < .001, Cohen's *d* = .64; bully: *M* = 3.10, *p* < .001, Cohen's *d* = .81; pro‐bully: *M* = 3.07, *p* < .001, Cohen's *d* = .75; bystander: *M* = 1.81, *p* < .001, Cohen's *d* = 1.40). The defender, the bully and the pro‐bully showed higher visit counts compared with the bystander (*p* < .001 for all the comparisons, Cohen's *d* = 1.23, .90 and .82).

### Total fixation durations

The third attentional index was the total fixation durations for each AOI (Table 3). In the physical bullying scenario, a significant role effect was found, *F(*1,49) = 39.09, *η*_*p*_^*2*^ = .44, *p* < .001 (Table 3). Bonferroni showed that the victim (*M* = 1.97) presented a longer time of fixations with medium effect size compared with the bully (*M* = 1.56, *p* = .003, Cohen's *d* = .56). The victim and the bully presented a longer total fixation durations compared with all the other roles (defender: *M* = 1.21, *p* < .001 and *p* = .011, Cohen's *d* = 1.02 and .49; bystander: *M* = .99, *p* < .001 for both the comparisons, Cohen's *d* = .90 and .68; pro‐bully: *M* = .66, *p* < .001 for both the comparisons, Cohen's *d* = 1.24 and 1.20). Bystanders and defenders were fixed for longer periods of time than pro‐bullies (*p* = .005 and *p* < .001, Cohen's *d* = .53 and .84).

**TABLE 3:** Means, standard deviations and one‐way analyses of variance in total fixation duration among roles in different forms of bullying and cyberbullying.

	Bully (A)		Victim (B)		Pro‐bully (C)		Defender (D)		Bystander (E)		F (1,49)	Bonferroni post‐hoc
	*M*	*SD*	*M*	*SD*	*M*	*SD*	*M*	*SD*	*M*	*SD*		
Physical bullying	1.56	.95	1.97	1.22	.66	.73	1.21	.88	.99	.86	39.09***	B > A A,B > C,D,E D,E > C
Verbal bullying	1.24	.98	1.44	1.00	.67	.66	.78	.55	.94	.61	16.51***	A,B > C,D B > E E > C
Relational bullying	1.54	1.10	1.52	1.01	.82	.72	.75	.62	1.03	.60	23.00***	A,B > C,D,E E > D
Cyberbullying	1.78	1.08	1.97	1.18	1.14	.86	1.37	.80	.93	.64	27.69***	A,B > C,D,E D > E

*** p < .001.

With regard to verbal bullying, a significant role effect was confirmed, *F*(1,49) = 16.51, *η*_*p*_^*2*^ = .25, *p* < .001 (Table 3). Bonferroni post‐hoc analysis revealed that the victim (*M* = 1.44) and the bully (*M* = 1.24) showed the longest time of fixations compared with the defender (*M* = .78, *p* < .001 and *p*=. 004, Cohen's *d* = .80 and .53) and the pro‐bully (*M* = .67, *p* < .001 for both the comparisons, Cohen's *d* = .89 and .59). In addition, the victim was watched for a significantly longer period of time compared with the bystander (*M* = .94, *p* < .001, Cohen's *d* = .68). Finally, the bystander received a longer fixation compared with the pro‐bully (*p* = .048, Cohen's *d* = .42).

The relational bullying showed a significant role effect, *F*(1,49) = 23.00, *η*_*p*_^*2*^ = .32, *p* < .001 (Table 3). Again the victim (*M* = 1.52) and the bully (*M* = 1.54) presented the longest total fixation with a significant difference and large effect sizes compared with the bystander (*M* = 1.03, *p* < .001 and *p* = .008, Cohen's *d* = .55 and .51), the pro‐bully (*M* = .82, *p* < .001 for both the comparisons, Cohen's *d* = .92 and .95) and the defender (*M* = .75, *p* < .001 for both the comparisons, Cohen's *d* = 1.04 and .93) and the bystander presented longer fixations compared with the defender (*p* = .02, Cohen's *d* = .47).

Cyberbullying also confirmed a significant role effect, *F*(1,49) = 27.69, *η*_*p*_^*2*^ = .36, *p* < .001 (Table 3). Bonferroni post‐hoc showed that the victim (*M* = 1.97) and the bully (*M* = 1.78) attracted the longest duration of time with large and medium effect sizes compared with the other roles (defender: *M* = 1.37, *p* < .001 and *p* = .002, Cohen's *d* = .89 and .46; pro‐bully: *M* = 1.14, *p* < .001 for both the comparisons, Cohen's *d* = .94 and .75; bystander: *M* = .93, *p* < .001 for both the comparisons, Cohen's *d* = 1.16 and 1.00). The defender showed a longer time of fixations than the bystander (*p* < .001, Cohen's *d* = .63).

### Interview data

Overall, the bully and the victim were the roles most frequently recognized and described at almost 100% for all the vignettes.

In physical bullying, Cochran's Q test indicated a significant difference among the roles, *χ* ^*2*^(4) = 173.54, *p* < .001 (Table 4). Pairwise comparisons showed no significant difference between the bully and the victim (*p* = 1.00), while they were significantly more mentioned than the other roles (*p* < .001 for all the comparisons). No significant difference was found between the defender and the pro‐bully (*p* = 1.00). In addition, the defender and the pro‐bully received more mentions than the bystander (*p* < .001 for both comparisons).

**TABLE 4:** Numbers, Percentages and cochran's Q test among roles in different forms of bullying and cyberbullying.

	Bully (A)		Victim (B)		Pro‐bully (C)		Defender (D)		Bystander (E)		*χ* ^*2*^(4)	Pairwise multiple comparisons
	*N*	%	*N*	%	*N*	%	*N*	%	*N*	%		
Physical bullying	150	100%	150	100%	119	79%	106	73%	75	50%	173.54***	A,B > C,D,E C,D > E
Verbal bullying	150	100%	150	100%	105	70%	77	51%	70	47%	218.20***	A,B > C,D,E C > D,E
Relational bullying	145	97%	148	99%	101	67%	82	55%	58	39%	225.80***	A,B > C,D,E C > D,E D > E
Cyberbullying	136	91%	127	85%	110	73%	100	67%	69	46%	129.54***	A,B > C,D,E C,D > E

*Note*: Each student described three vignettes for each type of bullying and cyberbullying.

Concerning verbal bullying, Cochran's Q test found a significant difference among the roles, *χ* ^2^(4) = 218.20, *p* < .001 (Table 4). Pairwise comparisons confirmed the lack of significant difference between the bully and the victim (*p* = 1.00), while the bully and the victim were more identified in comparison with the other roles (*p* < .001 for all the comparisons). In addition, the pro‐bully was significantly mentioned more than the defender and the bystander (*p* < .001 for both comparisons), while no difference was found between them (*p* = .34).

In relational bullying, Cochran's Q test showed a significant difference *χ* ^2^(4) = 225.80, *p* < .001 (Table 4). Pairwise comparisons revealed no significant difference between the bully and the victim (*p* = .69), while they were more identified compared with the other roles (*p* < .001 for all the comparisons). Moreover, the pro‐bully was more identified than the defender (*p* = .01) and the bystander (*p* < .001) and the defender compared with the bystander (*p* < .001).

Also in cyberbullying, Cochran's Q test showed a significant difference *χ* ^2^(4) = 129.54, *p* < .001 (Table 4). Pairwise comparisons revealed no significant difference between the bully and the victim (*p* = .170) and between the pro‐bully and the defender (*p* = .122). The bully and the victim were more identified compared with the other roles (*p* < .001 for all the comparisons; for the comparison between the victim and the pro‐bully, *p* = .009). Finally, the defender and the pro‐bully were more identified compared with the bystander (*p* < .001 for both comparisons).

## DISCUSSION

In the present study, we investigated whether the eye‐tracker could provide insights into students' perceptions of roles in bullying and cyberbullying. We also analysed data from student interviews to understand salience in role recognition and verify the roles most correctly identified. Our results highlight that bullying and cyberbullying phenomena are mainly perceived as dyadic among students, confirming findings from previous studies (Bosacki et al., 2006; Guarini et al., 2019; Rigby, 2020; Thornberg & Knutsen, 2011). Indeed, the victim and the bully represented the most observed and identified roles compared with the other roles in the different bullying (physical, verbal, relational) and cyberbullying scenarios. Overall, we surmise that the differences between the dyad and the other roles could be attributed to different attentional processes, namely bottom‐up and top‐down processes, as already described in the introduction. Indeed, although the use of bottom‐up and top‐down processes has been studied primarily on non‐social stimuli, studies suggest that these processes can also drive attention in social interaction (Flechsenhar et al., 2018). Similar considerations emerged from the Social Information Processing Model (Crick & Dodge, 1994) as it revealed that the first stages of processing social information involve encoding and interpreting the important primary cues to decide what is the most appropriate behaviour in response to those social cues. Therefore, it appears reasonable that the bully and the victim can attract a bottom‐up type of attention, helping students to immediately understand what is happening. In other words, to interpret what is happening, it is more salient to look at those who commit or suffer the aggressive action than those who do not participate directly. By contrast, the attention to the other roles suggested top‐down process in which participants tried to interpret the other roles based on their motivation, a‐priori knowledge and experiences.

Regarding physical bullying, the victim and the bully were the most observed and correctly named roles across all the metrics, confirming a bottom‐up process. Concerning eye movements, within the dyad, results showed that the victim received more fixations with a longer duration than the bully, meaning that students explored the victim longer and with more attention than the bully, probably because he/she received direct and physical aggression. The interviews did not show any differences between victims and bullies, as both roles were identified by all students. Outside the roles of victim and bully, eye movements revealed that the defender and the bystander were watched significantly more than the pro‐bully across all measures. This difference could be explained by the different role functions. Indeed, the defenders can disrupt the aggression, increasing the attention a student pays to this role and for a longer period than pro‐bullies who reinforce already established behaviours. Concerning the bystander, previous research has shown that this role does not often receive fair representation as playing an important role in bullying or cyberbullying. Khanolainen and Semenova (2020) and Warwick and Purdy (2019) found that the bystander was never considered important by students. In our study, we surmised that the greater attention paid to the bystander compared with the pro‐bully might be due to the effort of the observer in combining their a priori knowledge about these dynamics with the contextual information of the drawings to understand the bystander's intention and function in the scenes. In other words, we think that students recognized that the bystander was part of the scene; however, their function was not immediately as clear as the other roles. Therefore, the greater focus suggests an effort to determine the nature of the bystander role. A different trend emerged from the interviews as students were more likely to mention the pro‐bully and the defender than the bystander, probably because their function within these dynamics is ‘active’ and they commented or completed an action during the aggression. In contrast, a bystander is simply an observer with a neutral expression and for this reason, a large majority of students did not mention this, even if they took the time to investigate it. Consequently, the students were attentive to the bystander implicitly, but this role did not seem to be as crucial when the vignette had to be described verbally.

Regarding verbal bullying, our results showed that the bully and the victim received a greater number of fixation and visit counts compared with all the other roles. Likewise, results from interviews showed a 100% recognition rate for both victims and bullies. Concerning the other roles, eye‐movements suggested that the fixation durations for the victim and bully were longer than those for the defender and pro‐bully, but only the victim was fixed longer than the bystander. This finding aligns with the study by Bosacki et al. (2006), where they asked students of different ages (8–12 years) to draw some bullying scenes freely. They found that most of the 11–12‐year‐old students who depicted verbal bullying just drew the ‘bully‐victim’ dyad. We also found that the bystander presented a greater number of visit counts than the defender, indicating that students returned more often to the role of the bystander. Moreover, the bystander received a greater number of fixation counts and a longer fixation compared with the pro‐bully, confirming greater attention towards the bystander. As we have suggested, the increased focus on the bystander testifies to a top‐down mechanism in which students need to explore more of the bystander's role to understand their function in the scenario. Indeed, as other research has suggested, the way in which bystanders respond when witnessing bullying influences the extent to which bullying behaviour takes place (Gini et al., 2008). The bystander can decide to ignore the situation, remain passive and, thus, reinforce the victimization, or they can choose to join the defender, and, therefore, stop the aggression (Salmivalli, 1999). Noteworthy is that in this type of bullying, both the defender and bystander's identification were comparable, whereas the pro‐bully's recognition rate was considerably higher. The lack of description for the bystander could be explained, as already stated, by the fact that the bystander has no clearly identified function and it was not active in the vignette. Concerning the defender, interviews confirmed results obtained from the eye‐tracker with an underrepresentation of this role for verbal bullying. Indeed, as suggested by Bauman et al. (2020), the choice to intervene to stop school bullying was less likely in verbal and social bullying compared with physical bullying. It was hypothesized that because these types of bullying are so prevalent and normalized, peers lack the motivation to intervene, thereby decreasing the importance of the defender. This may have been reflected not only in the less attention given to it but also in the lack of its description during interviews.

Concerning relational bullying, we found that the victim and the bully showed higher scores for all three attentional indices compared with other roles, in line with the previous types of bullying and confirming their predominance in the scenes as well as the bottom‐up process. These findings were also confirmed by the analysis of the interviews, which showed a high percentage of recognition ranging from 97% to 99%. Regarding the other roles involved, eye movements revealed only a difference in the total fixation duration between the bystander and the defender. The bystander, once again, was observed longer. Similar to physical and verbal bullying, we believe that the increased focus on the bystander represented the effort of the individual to interpret the role. Considering the findings from the interviews, we found that the pro‐bully was mentioned more often than the defender and bystander while the defender was mentioned more than the bystander. As already proposed for verbal bullying, the capacity of students to recognize the important role of the defender in the social situation could be minimized compared with the pro‐bully as these types of situations could not be perceived as circumstances that require help. The role of the bystander does not appear important enough to be mentioned as it has already happened for the other types of bullying.

Regarding cyberbullying, we found that the bully and the victim were more observed than the others in the fixation counts and total duration fixation. Concerning the interviews, in spite of being the most mentioned roles, the recognition rates were slightly below compared with the other types of bullying, ranging from 85% to 91%. It is possible that students showed a lower identification rate for the victim and the bully as the aggressive action was mediated by the use of technologies (e.g. a smartphone) without visual contact between the bully and the victim. Concerning visit counts, the victim received a greater number of visits than the bully and the other roles, suggesting students felt the need to enter the victim's area of interest more often, probably to observe the victim's relationship with the other roles. In addition, the defender received greater fixation counts and visit counts compared with the bystander. Looking at the students' descriptions, the defender and the pro‐bully appeared to be recognized more often than the bystander, which does not seem to play an important role requiring a mention.

In conclusion, our results confirmed that bullying and cyberbullying are considered mainly dyadic phenomena, given the great importance of the bully and the victim, as shown by both implicit and explicit measures. Various factors may contribute to students' focus on the bully and the victim, including narrations of films and fairy tales with often are based on a protagonist and antagonist (Gupta & Yilmaz, 2018); this may lead young students to pay more attention to the two main characters in a scene.

Regarding the other roles, we found a discrepancy between explicit and implicit measures. Indeed, while the pro‐bully, overall, seems to receive less attention, it is one of the most mentioned roles following the bully and the victim. The pro‐bully may be given less attention than other characters because its function is easier to understand since it reinforces a behaviour. However, for its role in reinforcing behaviour, it is more likely to be mentioned as part of the scenes. The defender captured more attention in physical bullying and cyberbullying, while it seems less important in verbal bullying. The analysis of the interview confirmed this trend also at an explicit level; as already stated, it is possible that these types of bullying are so prevalent that students do not pay attention to the defender and do not mention it since they do not think that the intervention is necessary. Finally, the bystander is perhaps the role with the greatest discrepancy between implicit and explicit measurements. Overall, a greater focus on the bystander in physical, verbal and relational bullying was found and attributed to a greater effort of interpretation required, while the analysis of the interviews revealed that the bystander received the least mentions, probably because it was not considered as an important part of the vignette.

### Limitations and further research

Although this study is an important step in understanding the attention paid to different roles in bullying and cyberbullying, it is not without shortcomings. All the students were aged 10 to 11 years old. While physical, relational and verbal bullying are phenomena already present during the primary school years (Pouwels et al., 2018), cyberbullying is more typical among older students (DeSmet et al., 2018). It would be worthwhile to understand how cyberbullying is observed by older students who, potentially, could be more involved in these dynamics and whether they would have similar responses. In addition, even though cyberbullying has received much attention over the past decade, there is still a lack of consensus on exactly how cyberbullying should be conceptualized (Scheithauer et al., 2021). Compared with face‐to‐face bullying, cyberbullying can happen both in the online environment, for example, using social networks (Kowalski et al., 2019) and in the physical environment (e.g. filming a physical attack on a victim with the aim of distributing it via the internet) (Kowalski et al., 2008; Kowalski & Limber, 2013). In our study, we considered cyberbullying in the physical environment, and we acknowledge that the behaviour of the cyberbullying roles could change in an online environment (Scheithauer et al., 2021).

The second limitation concerns the proposed stimuli to the students, which pose three elements of attention. The first element of attention is the use of drawings instead of real video scenes with human actors. Indeed, using human actors in pictures or movies can increase ecological validity compared with using cartoon characters (Riby & Hancock, 2009). However, we chose drawings as the stimuli to remove some of the socially demanding factors associated with human interactions while representing real and possible scenarios among people (Riby & Hancock, 2009). Moreover, as discussed above, it is necessary to be cautious in the interpretation of data, keeping in mind that our study was exploratory, and the generalizability of our findings is limited by the unique methodology used (Vraga et al., 2016). Indeed, despite using interviews to understand better the perceived salience and importance given to different roles, we cannot exclude that other types of variables and factors may intervene. The second element is the use of a single character for each role. We chose not to include so many characters in the same vignettes; however, this point could be particularly critical, especially for bystanders, as in real life, more students acted together in the same role. The last point regarding the stimuli was the position within the vignettes of each character's role. Even if we tried placing characters in different portions of the scene among vignettes, a complete balance was not possible. Further studies could confirm our preliminary results using new stimuli such as video, including groups for each role, and balancing the position of each character's role.

Third, it is important to understand whether particular levels of victimization and/or bullying might influence how students observe these scenes. Although the use of drawings instead of real images allowed us to avoid the drawbacks (e.g. the emotional appeal of images), we cannot rule out that particularly severe experiences of victimization affected the patterns of observation. Furthermore, using self‐report questionnaires to evaluate the intensity of bullying and cyberbullying and the role each student plays in these dynamics would be extremely valuable, as this would allow us to understand whether different roles correlate with different types of observations.

## CONCLUSIONS AND IMPLICATIONS FOR INTERVENTIONS

To the best of our knowledge, this is the first study to investigate the role perceptions in bullying and cyberbullying using explicit and implicit measures. The results of the study underscore the importance of exploring the two phenomena by expanding and integrating the use of new methodologies, such as the eye‐tracker. In this way, it is possible to increase our knowledge of the phenomena and address more effective interventions. Indeed, we believe understanding how attention is paid when observing the behaviour of others is important for the subsequent processing of information and the way we intervene. In this regard, the overall inattention to other roles present in the scene may partly be responsible for the failure to intervene to stop the aggression, especially in verbal and relational bullying where the defender seems less important. Furthermore, it is essential to consider the roles that are not mentioned during interviews, such as bystanders. Overall, these findings indicate that further research on group dynamics is necessary to promote social and non‐dyadic views in interpreting phenomena and increase social responsibility. In their meta‐analysis, Gaffney et al. (2019) found that peer involvement components (e.g. group discussions or role‐playing activities and encouraging bystanders to intervene) were associated with greater overall effectiveness of the interventions. Based on our findings, as part of any intervention, peers may also benefit from training in their capacity to detect significant and threatening cues in bullying and cyberbullying episodes. In this regard, combining all the strategies and activities already used with training based on eye‐tracking could be interesting, perhaps showing them how their attention works during these episodes.

Finally, it could be worthwhile to use the eye‐tracker as a tool to assess the efficacy of the interventions. For example, to evaluate whether the students' attentional pattern might have changed after receiving an intervention to prevent bullying and cyberbullying, with particular attention to the role of the defender and the bystander, and whether this might instead have remained unchanged in the control group. Greater attention given to the defender and the bystander might testify to a greater awareness of the importance of these roles and a first step to promoting responsibility among students.

## AUTHOR CONTRIBUTIONS

**Laura Menabò:** Conceptualization; formal analysis; methodology; writing – original draft; writing – review and editing. **Grace Skrzypiec:** Writing – review and editing. **Phillip Slee:** Writing – review and editing. **Annalisa Guarini:** Conceptualization; methodology; writing – review and editing.

## CONFLICT OF INTEREST STATEMENT

All authors declare no conflict of interest.

## Supporting information


Data S1:

## Data Availability

The data that support the findings of this study are available from the corresponding author upon reasonable request.

## References

[bjep12604-bib-1001] Aricak, T. , Siyahhan, S. , Uzunhasanoglu, A. , Saribeyoglu, S. , Ciplak, S. , Yilmaz, N. , & Memmedov, C. (2008). Cyberbullying among Turkish Adolescents. CyberPsychology & Behavior, 11(3), 253–261. 10.1089/cpb.2007.0016 18537493

[bjep12604-bib-0002] Arseneault, L. (2018). Annual research review: The persistent and pervasive impact of being bullied in childhood and adolescence: Implications for policy and practice. Journal of Child Psychology and Psychiatry, 59(4), 405–421. 10.1111/jcpp.12841 29134659 PMC6542665

[bjep12604-bib-0004] Bauman, S. , Yoon, J. , Iurino, C. , & Hackett, L. (2020). Experiences of adolescent witnesses to peer victimization: The bystander effect. Journal of School Psychology, 80, 1–14. 10.1016/j.jsp.2020.03.002 32540087

[bjep12604-bib-1002] Berne, S. , Frisén, A. , Schultze‐Krumbholz, A. , Scheithauer, H. , Naruskov, K. , Luik, P. , Katzer, C. , Erentaite, R. , & Zukauskiene, R. (2013). Cyberbullying assessment instruments: A systematic review. Aggression and Violent Behavior, 18(2), 320–334. 10.1016/j.avb.2012.11.022

[bjep12604-bib-0005] Bosacki, S. , Marini, Z. , & Dane, A. (2006). Voices from the classroom: Pictorial and narrative representations of children's bullying experiences. Journal of Moral Education, 35(2), 231–245. 10.1080/03057240600681769

[bjep12604-bib-0006] Burton, K. A. , Florell, D. , & Wygant, D. B. (2013). The role of peer attachment and normative beliefs about aggression on traditional bullying and cyberbullying: Peer attachment, aggression, and cyberbullying. Psychology in the Schools, 50(2), 103–115. 10.1002/pits.21663

[bjep12604-bib-0007] Buschman, T. J. , & Miller, E. K. (2007). Top‐down versus bottom‐up control of attention in the prefrontal and posterior parietal cortices. Science, 315(5820), 1860–1862. 10.1126/science.1138071 17395832

[bjep12604-bib-0008] Campbell, M. , & Bauman, S. (2018). Cyberbullying: Definition, consequences, prevalence. In Reducing cyberbullying in schools (pp. 3–16). Elsevier. 10.1016/B978-0-12-811423-0.00001-8

[bjep12604-bib-0009] Caravita, S. C. , Colombo, B. , Stefanelli, S. , & Zigliani, R. (2016). Emotional, psychophysiological and behavioral responses elicited by the exposition to cyberbullying situations: Two experimental studies. Psicología Educativa, 22(1), 49–59.

[bjep12604-bib-0010] Casper, D. M. (2021). Types of traditional (offline) bullying. In P. K. Smith & J. O. Norman (Eds.), The Wiley Blackwell handbook of bullying: A comprehensive and international review of research and intervention (pp. 96–119). Wiley Blackwell. 10.1002/9781118482650.ch6

[bjep12604-bib-0011] Connor, C. E. , Egeth, H. E. , & Yantis, S. (2004). Visual attention: Bottom‐up versus top‐down. Current Biology, 14(19), R850–R852. 10.1016/j.cub.2004.09.041 15458666

[bjep12604-bib-0012] Corbetta, M. , & Shulman, G. L. (2002). Control of goal‐directed and stimulus‐driven attention in the brain. Nature Reviews Neuroscience, 3(3), 201–215. 10.1038/nrn755 11994752

[bjep12604-bib-0013] Corbetta, P. (2003). Social research: Theory, methods and techniques. Sage.

[bjep12604-bib-0014] Cornell, D. G. , & Bandyopandhyay, S. (2010). Assessment of bullying. In S. R. Jimerson , S. M. Swearer , & D. L. Espelage (Eds.), Handbook of bullying in schools: An international perspective (pp. 265–276). Routledge.

[bjep12604-bib-0016] Creswell, J. (2013). Qualitative inquiry and research design: Choosing among five approaches (3rd ed.). Sage.

[bjep12604-bib-0017] Crick, N. R. , & Dodge, K. A. (1994). A review and reformulation of social information‐processing mechanisms in children's social adjustment. Psychological Bulletin, 115(1), 74–101.

[bjep12604-bib-0018] Cross, D. , Barnes, A. , Papageorgiou, A. , Hadwen, K. , Hearn, L. , & Lester, L. (2015). A social–ecological framework for understanding and reducing cyberbullying behaviours. Aggression and Violent Behavior, 23, 109–117. 10.1016/j.avb.2015.05.016

[bjep12604-bib-0019] DeCoster, J. , Banner, M. J. , Smith, E. R. , & Semin, G. R. (2006). On the inexplicability of the implicit: Differences in the information provided by implicit and explicit tests. Social Cognition, 24(1), 5–21. 10.1521/soco.2006.24.1.5

[bjep12604-bib-0020] Dennehy, R. , Meaney, S. , Walsh, K. A. , Sinnott, C. , Cronin, M. , & Arensman, E. (2020). Young people's conceptualizations of the nature of cyberbullying: A systematic review and synthesis of qualitative research. Aggression and Violent Behavior, 51, 101379. 10.1016/j.avb.2020.101379

[bjep12604-bib-0021] Desimone, R. , & Duncan, J. (1995). Neural mechanisms of selective visual attention. Annual Review of Neuroscience, 18(1), 193–222. 10.1146/annurev.ne.18.030195.001205 7605061

[bjep12604-bib-0022] DeSmet, A. , Rodelli, M. , Walrave, M. , Soenens, B. , Cardon, G. , & De Bourdeaudhuij, I. (2018). Cyberbullying and traditional bullying involvement among heterosexual and non‐heterosexual adolescents, and their associations with age and gender. Computers in Human Behavior, 83, 254–261. 10.1016/j.chb.2018.02.010

[bjep12604-bib-1003] Dys, S. P. (2019). How Children Cognitively Process and Emotionally Respond to Victimizing Others: A Multimethod Developmental Approach . Doctoral dissertation. University of Toronto (Canada).

[bjep12604-bib-0024] Espelage, D. L. , & Swearer, S. M. (2003). Research on school bullying and victimization: What have we learned and where do we go from here? School Psychology Review, 32(3), 365–383. 10.1080/02796015.2003.12086206

[bjep12604-bib-0025] Flechsenhar, A. , Rösler, L. , & Gamer, M. (2018). Attentional selection of social features persists despite restricted bottom‐up information and affects temporal viewing dynamics. Scientific Reports, 8, 12555. 10.1038/s41598-018-30736-8 30135443 PMC6105690

[bjep12604-bib-1004] Frey, L. R. , Botan, C. H. , & Kreps, G. L. (2000). Investigating Communication: An Introduction to Research Methods (2nd ed.). Allyn and Bacon.

[bjep12604-bib-0026] Gaffney, H. , Ttofi, M. M. , & Farrington, D. P. (2019). Evaluating the effectiveness of school‐bullying prevention programs: An updated meta‐analytical review. Aggression and Violent Behavior, 45, 111–133. 10.1016/j.avb.2018.07.001

[bjep12604-bib-0027] Giacomantonio, M. , Jordan, J. , Federico, F. , van den Assem, M. J. , & van Dolder, D. (2018). The evil eye: Eye gaze and competitiveness in social decision making: Eye‐gaze & competition. European Journal of Social Psychology, 48(3), 388–396. 10.1002/ejsp.2336

[bjep12604-bib-0028] Giesbrecht, A. L. (2019). What eye tracking reveals in implicit‐discrete versus explicit‐continuous theory‐of‐mind measures. Kwantlen Psychology Student Journal, 1, 1–46.

[bjep12604-bib-0029] Gini, G. , Pozzoli, T. , Borghi, F. , & Franzoni, L. (2008). The role of bystanders in students' perception of bullying and sense of safety. Journal of School Psychology, 46(6), 617–638. 10.1016/j.jsp.2008.02.001 19083376

[bjep12604-bib-0030] Gini, G. , Pozzoli, T. , Jenkins, L. , & Demaray, M. (2021). Participant roles in bullying. In P. K. Smith & J. O. Norman (Eds.), The Wiley Blackwell handbook of bullying (1st ed., pp. 76–95). Wiley. 10.1002/9781118482650.ch5

[bjep12604-bib-1005] Graham, D. J. , Orquin, J. L. , & Visschers, V. H. M. (2012). Eye tracking and nutrition label use: A review of the literature and recommendations for label enhancement. Food Policy, 37(4), 378–382. 10.1016/j.foodpol.2012.03.004

[bjep12604-bib-0032] Guarini, A. , Menin, D. , Menabò, L. , & Brighi, A. (2019). RPC teacher‐based program for improving coping strategies to Deal with cyberbullying. International Journal of Environmental Research and Public Health, 16(6), 948. 10.3390/ijerph16060948 30884790 PMC6466270

[bjep12604-bib-0033] Guarini, A. , Tobia, V. , Bonifacci, P. , Faldella, G. , & Sansavini, A. (2021). Magnitude comparisons, number knowledge and calculation in VeryPreterm children and children with specific learning disability: A Cross‐population study using eye‐tracking. Journal of Learning Disabilities, 54(2), 83–96. 10.1177/0022219420950651 32814504

[bjep12604-bib-1006] Gupta, A. , & Yilmaz, A. (2018). Social network inference in videos. Academic Press Library in Signal Processing, 6, 395–424. 10.1016/b978-0-12-811889-4.00011-7

[bjep12604-bib-0034] Guzman‐Holst, C. , & Bowes, L. (2021). Bullying and internalizing symptoms. In P. K. Smith & J. O. Norman (Eds.), The Wiley Blackwell handbook of bullying: A comprehensive and international review of research and intervention (pp. 561–579). Wiley Blackwell.

[bjep12604-bib-0035] Haataja, A. , Ahtola, A. , Poskiparta, E. , & Salmivalli, C. (2015). A process view on implementing an antibullying curriculum: How teachers differ and what explains the variation. School Psychology Quarterly, 30(4), 564–576. 10.1037/spq0000121 25893281

[bjep12604-bib-0036] Hase, C. N. , Goldberg, S. B. , Smith, D. , Stuck, A. , & Campain, J. (2015). Impacts of traditional bullying and cyberbullying on the mental health of middle school and high school students: Impacts of traditional bullying and cyberbullying. Psychology in the Schools, 52(6), 607–617. 10.1002/pits.21841

[bjep12604-bib-0037] Hemphill, S. A. , Tollit, M. , Kotevski, A. , & Heerde, J. A. (2015). Predictors of traditional and cyber‐bullying victimization: A longitudinal study of Australian secondary school students. Journal of Interpersonal Violence, 30(15), 2567–2590. 10.1177/0886260514553636 25315480

[bjep12604-bib-0038] Hinduja, S. , & Patchin, J. W. (2010). Bullying, cyberbullying, and suicide. Archives of Suicide Research, 14(3), 206–221. 10.1080/13811118.2010.494133 20658375

[bjep12604-bib-0039] Hunter, S. C. , Noret, N. , & Boyle, J. M. E. (2021). Measurement Issues Relevant to Questionnaire Data. In P. K. Smith & J. O. Norman (Eds.), The Wiley Blackwell handbook of bullying (1st ed., pp. 178–195). Wiley. 10.1002/9781118482650.ch10

[bjep12604-bib-0040] Hutson, E. , Kelly, S. , & Militello, L. K. (2018). Systematic review of cyberbullying interventions for youth and parents with implications for evidence‐based practice: Cyberbullying interventions for individual youth and parents. Worldviews on Evidence‐Based Nursing, 15(1), 72–79. 10.1111/wvn.12257 28859246 PMC8074991

[bjep12604-bib-0041] Hymel, S. , Wagner, E. , & Butler, L. J. (1990). Reputational bias: View from the peer group. In S. R. Asher & J. D. Coie (Eds.), Peer rejection in childhood (pp. 156–186). Cambridge University Press.

[bjep12604-bib-0042] Itti, L. , & Koch, C. (2001). Feature combination strategies for saliency‐based visual attention systems. Journal of Electronic Imaging, 10(1), 161. 10.1117/1.1333677

[bjep12604-bib-0043] Jungert, T. , Piroddi, B. , & Thornberg, R. (2016). Early adolescents' motivations to defend victims in school bullying and their perceptions of student–teacher relationships: A self‐determination theory approach. Journal of Adolescence, 53(1), 75–90. 10.1016/j.adolescence.2016.09.001 27654402

[bjep12604-bib-0044] Just, M. A. , & Carpenter, P. A. (1976). The role of eye‐fixation research in cognitive psychology. Behavior Research Methods & Instrumentation, 8(2), 139–143. 10.3758/BF03201761

[bjep12604-bib-0045] Katsuki, F. , & Constantinidis, C. (2014). Bottom‐up and top‐down attention: Different processes and overlapping neural systems. The Neuroscientist, 20(5), 509–521. 10.1177/1073858413514136 24362813

[bjep12604-bib-0046] Khanolainen, D. , & Semenova, E. (2020). School bullying through graphic vignettes: Developing a new arts‐based method to study a sensitive topic. International Journal of Qualitative Methods, 19, 160940692092276. 10.1177/1609406920922765

[bjep12604-bib-0047] Klomek, A. B. , Sourander, A. , & Gould, M. (2010). The Association of Suicide and Bullying in childhood to young adulthood: A review of Cross‐sectional and longitudinal research findings. The Canadian Journal of Psychiatry, 55(5), 282–288. 10.1177/070674371005500503 20482954

[bjep12604-bib-0048] Koornneef, A. , & Vanberkum, J. (2006). On the use of verb‐based implicit causality in sentence comprehension: Evidence from self‐paced reading and eye tracking. Journal of Memory and Language, 54(4), 445–465. 10.1016/j.jml.2005.12.003

[bjep12604-bib-0049] Kowalski, R. M. , Giumetti, G. W. , Schroeder, A. N. , & Lattanner, M. R. (2014). Bullying in the digital age: A critical review and meta‐analysis of cyberbullying research among youth. Psychological Bulletin, 140(4), 1073–1137. 10.1037/a0035618 24512111

[bjep12604-bib-0050] Kowalski, R. M. , & Limber, S. P. (2013). Psychological, physical, and academic correlates of cyberbullying and traditional bullying. Journal of Adolescent Health, 53(1), S13–S20. 10.1016/j.jadohealth.2012.09.018 23790195

[bjep12604-bib-0051] Kowalski, R. M. , Limber, S. P. , & Agatston, P. W. (2008). Cyber bullying: Bullying in the digital age. Blackwell Publishing.

[bjep12604-bib-1007] Kowalski, R. M. , Limber, S. P. , & McCord, A. (2019). A developmental approach to cyberbullying: Prevalence and protective factors. Aggression and Violent Behavior, 45, 20–32. 10.1016/j.avb.2018.02.009

[bjep12604-bib-0052] Koyanagi, A. , Oh, H. , Carvalho, A. F. , Smith, L. , Haro, J. M. , Vancampfort, D. , … DeVylder, J. E. (2019). Bullying victimization and suicide attempt among adolescents aged 12–15 years from 48 countries. Journal of the American Academy of Child & Adolescent Psychiatry, 58(9), 907–918. 10.1016/j.jaac.2018.10.018 30926574

[bjep12604-bib-0054] Mameli, C. , Menabò, L. , Brighi, A. , Menin, D. , Culbert, C. , Hamilton, J. , Scheithauer, H. , Smith, P. K. , Völlink, T. , Willems, R. A. , Purdy, N. , & Guarini, A. (2022). Stay safe and strong: Characteristics, roles and emotions of student‐produced comics related to cyberbullying. International Journal of Environmental Research and Public Health, 19(14), 8776. 10.3390/ijerph19148776 35886631 PMC9324025

[bjep12604-bib-0055] Mancas, M. (2009). Relative influence of bottom‐up and top‐down attention. Attention in Cognitive Systems, Lecture Notes in Computer Science, 5395, 212–226.

[bjep12604-bib-0056] Maran, D. A. , & Begotti, T. (2021). Measurement issues relevant to qualitative studies. In P. K. Smith & J. O. Norman (Eds.), The Wiley Blackwell handbook of bullying (1st ed., pp. 233–249). Wiley. 10.1002/9781118482650.ch13

[bjep12604-bib-0057] McConnell, L. , & Troop‐Gordon, W. (2021). Attentional biases to bullies and bystanders and Youth's coping with peer victimization. The Journal of Early Adolescence, 41(1), 97–127. 10.1177/0272431620931206

[bjep12604-bib-0058] Menesini, E. , & Nocentini, A. (2009). Cyberbullying definition and measurement: Some critical considerations. Zeitschrift Für Psychologie / Journal of Psychology, 217(4), 230–232. 10.1027/0044-3409.217.4.230

[bjep12604-bib-0059] Mishna, F. , Wiener, J. , & Pepler, D. (2008). Some of my best friends – Experiences of bullying within friendships. School Psychology International, 29(5), 549–573. 10.1177/0143034308099201

[bjep12604-bib-0061] Nocentini, A. , Menesini, E. , & Salmivalli, C. (2013). Level and change of bullying behavior during high school: A multilevel growth curve analysis. Journal of Adolescence, 36(3), 495–505. 10.1016/j.adolescence.2013.02.004 23523327

[bjep12604-bib-0062] Oar, E. L. , Johnco, C. J. , Waters, A. M. , Fardouly, J. , Forbes, M. K. , Magson, N. R. , Richardson, C. E. , & Rapee, R. M. (2022). Eye‐tracking to assess anxiety‐related attentional biases among a large sample of preadolescent children. Behaviour Research and Therapy, 153, 104079. 10.1016/j.brat.2022.104079 35395478

[bjep12604-bib-1008] Olweus, D. (2012). Cyberbullying: An overrated phenomenon? European Journal of Developmental Psychology, 9(5), 520–538. 10.1080/17405629.2012.682358

[bjep12604-bib-1009] Patchin, J. W. , & Hinduja, S. (2006). Bullies move beyond the schoolyard. Youth Violence and Juvenile Justice, 4(2), 148–169. 10.1177/1541204006286288

[bjep12604-bib-0063] Pellegrini, A. D. , & Bartini, M. (2000). A longitudinal study of bullying, victimization, and peer affiliation during the transition from primary school to middle school. American Educational Research Journal, 37(3), 699–725. 10.3102/00028312037003699

[bjep12604-bib-0064] Pouwels, J. L. , van Noorden, T. H. J. , Lansu, T. A. M. , & Cillessen, A. H. N. (2018). The participant roles of bullying in different grades: Prevalence and social status profiles. Social Development, 27(4), 732–747. 10.1111/sode.12294

[bjep12604-bib-0065] Pozzoli, T. , & Gini, G. (2010). Active defending and passive Bystanding behavior in bullying: The role of personal characteristics and perceived peer pressure. Journal of Abnormal Child Psychology, 38(6), 815–827. 10.1007/s10802-010-9399-9 20228996

[bjep12604-bib-0066] Riby, D. M. , & Hancock, P. J. B. (2009). Do faces capture the attention of individuals with Williams syndrome or autism? Evidence from tracking eye movements. Journal of Autism and Developmental Disorders, 39, 421–431. 10.1007/s10803-008-0641-z 18787936

[bjep12604-bib-0067] Rigby, K. (2020). How do victims of bullying in Australian schools view their perpetrators – As individuals or as groups? Implications for educators. Australian Journal of Education, 64(1), 25–39. 10.1177/0004944119894099

[bjep12604-bib-0068] Rigby, K. , & Johnson, B. (2006). Expressed readiness of Australian schoolchildren to act as bystanders in support of children who are being bullied. Educational Psychology, 26(3), 425–440. 10.1080/01443410500342047

[bjep12604-bib-0069] Rivers, I. , & Smith, P. K. (1994). Types of bullying behaviour and their correlates. Aggressive Behavior, 20(5), 359–368. 10.1002/1098-2337(1994)20:5<359::AID-AB2480200503>3.0.CO;2-J

[bjep12604-bib-0070] Salmivalli, C. (1999). Participant role approach to school bullying: Implications for interventions. Journal of Adolescence, 22(4), 453–459. 10.1006/jado.1999.0239 10469509

[bjep12604-bib-1010] Salmivalli, C. (2010). Bullying and the peer group: A review. Aggression and Violent Behavior, 15(2), 112–120. 10.1016/j.avb.2009.08.007

[bjep12604-bib-1011] Salmivalli, C. , Lagerspetz, K. , Björkqvist, K. , Österman, K. , & Kaukiainen, A. (1996). Bullying as a group process: Participant roles and their relations to social status within the group. Aggressive Behavior, 22(1), 1–15. 10.1002/(sici)1098-2337(1996)22:1<1::aid-ab1>3.0.co;2-t

[bjep12604-bib-1012] Salmivalli, C. , Voeten, M. , & Poskiparta, E. (2011). Bystanders Matter: Associations Between Reinforcing, Defending, and the Frequency of Bullying Behavior in Classrooms. Journal of Clinical Child & Adolescent Psychology, 40(5), 668–676. 10.1080/15374416.2011.597090 21916686

[bjep12604-bib-0072] Scheithauer, H. , Schultze‐Krumbholz, A. , Pfetsch, J. , & Hess, M. (2021). Types of cyberbullying. In P. K. Smith & J. O. Norman (Eds.), The Wiley Blackwell handbook of bullying (1st ed., pp. 120–138). Wiley. 10.1002/9781118482650.ch7

[bjep12604-bib-1013] Seals, D. , & Young, J. (2003). Bullying and victimization: prevalence and relationship to gender, grade level, ethnicity, self‐esteem, and depression. Adolescence, 38(152), 735–747. 15053498

[bjep12604-bib-0073] Sigurdson, J.,. F. , Kaasbøll, J. , & Sund, A. M. (2021). Bullying and externalizing problems. In P. K. Smith & J. O. Norman (Eds.), The Wiley Blackwell handbook of bullying (1st ed, pp. 120–138). Wiley. 10.1002/9781118482650.ch7

[bjep12604-bib-0074] Silverman, D. (2013). Doing qualitative research: A practical handbook. Sage.

[bjep12604-bib-0075] Slonje, R. , Smith, P. K. , & Frisén, A. (2013). The nature of cyberbullying, and strategies for prevention. Computers in Human Behavior, 29(1), 26–32. 10.1016/j.chb.2012.05.024

[bjep12604-bib-0076] Smith, P. K. , del Barrio, C. , & Tokunaga, R. S. (2013). Definitions of bullying and cyberbullying: How useful are the terms? In S. Bauman , D. Cross , & J. Walker (Eds.), Principles of cyberbullying research: Definitions, measures, and methodology (pp. 26–40). Routledge/Taylor & Francis Group.

[bjep12604-bib-0077] Smith, P. K. , Robinson, S. , & Slonje, R. (2021). The school bullying research program: Why and how it has developed. In P. K. Smith & J. O. Norman (Eds.), The Wiley Blackwell handbook of bullying (1st ed., pp. 42–59). Wiley. 10.1002/9781118482650.ch3

[bjep12604-bib-0078] Stapinski, L. A. , Araya, R. , Heron, J. , Montgomery, A. A. , & Stallard, P. (2015). Peer victimization during adolescence: Concurrent and prospective impact on symptoms of depression and anxiety. Anxiety, Stress, & Coping, 28(1), 105–120. 10.1080/10615806.2014.962023 25214239

[bjep12604-bib-0079] Sumter, S. R. , Baumgartner, S. E. , Valkenburg, P. M. , & Peter, J. (2012). Developmental trajectories of peer victimization: Off‐line and online experiences during adolescence. Journal of Adolescent Health, 50(6), 607–613. 10.1016/j.jadohealth.2011.10.251 22626488

[bjep12604-bib-0081] Thornberg, R. , & Jungert, T. (2013). Bystander behavior in bullying situations: Basic moral sensitivity, moral disengagement and defender self‐efficacy. Journal of Adolescence, 36(3), 475–483. 10.1016/j.adolescence.2013.02.003 23522703

[bjep12604-bib-0082] Thornberg, R. , & Knutsen, S. (2011). Teenagers' explanations of bullying. Child & Youth Care Forum, 40(3), 177–192. 10.1007/s10566-010-9129-z

[bjep12604-bib-0083] Thornberg, R. , Wänström, L. , Hong, J. S. , & Espelage, D. L. (2017). Classroom relationship qualities and social‐cognitive correlates of defending and passive bystanding in school bullying in Sweden: A multilevel analysis. Journal of School Psychology, 63, 49–62. 10.1016/j.jsp.2017.03.002 28633938

[bjep12604-bib-0084] Tokunaga, R. S. (2010). Following you home from school: A critical review and synthesis of research on cyberbullying victimization. Computers in Human Behavior, 26(3), 277–287. 10.1016/j.chb.2009.11.014

[bjep12604-bib-1014] Troop‐Gordon, W. , Gordon, R. D. , Schwandt, B. M. , Horvath, G. A. , Ewing Lee, E. , & Visconti, K. J. (2019). Allocation of attention to scenes of peer harassment: Visual–cognitive moderators of the link between peer victimization and aggression. Development and Psychopathology, 31(2), 525–540. 10.1017/s0954579418000068 29562946 PMC6151173

[bjep12604-bib-0086] Volk, A. A. , Veenstra, R. , & Espelage, D. L. (2017). So you want to study bullying? Recommendations to enhance the validity, transparency, and compatibility of bullying research. Aggression and Violent Behavior, 36, 34–43. 10.1016/j.avb.2017.07.003

[bjep12604-bib-0087] Vraga, E. , Bode, L. , & Troller‐Renfree, S. (2016). Beyond self‐reports: Using eye tracking to measure topic and style differences in attention to social media content. Communication Methods and Measures, 10(2–3), 149–164. 10.1080/19312458.2016.1150443

[bjep12604-bib-0088] Warwick, D. , & Purdy, N. (2019). Cartoons as visual representations of the development of primary school children's understanding of bullying behaviours. Pastoral Care in Education, 37(3), 257–275. 10.1080/02643944.2019.1625430

[bjep12604-bib-0089] Weber, R. , Mangus, J. M. , & Huskey, R. (2015). Brain imaging in communication research: A practical guide to understanding and evaluating fMRI studies. Communication Methods and Measures, 9(1–2), 5–29. 10.1080/19312458.2014.999754

[bjep12604-bib-0090] Wolfe, J. M. (1994). Visual search in continuous, naturalistic stimuli. Vision Research, 34(9), 1187–1195. 8184562 10.1016/0042-6989(94)90300-x

[bjep12604-bib-0091] Xie, H. , & Ngai, S. S. (2020). Participant roles of peer bystanders in school bullying situations: Evidence from Wuhan, China. Children and Youth Services Review, 110, 104762. 10.1016/j.childyouth.2020.104762

[bjep12604-bib-1015] Zych, I. , Beltrán‐Catalán, M. , Ortega‐Ruiz, R. , & Llorent, V. J. (2018). Social and Emotional Competencies in Adolescents Involved in Different Bullying and Cyberbullying Roles. Revista de Psicodidáctica (English Ed.), 23(2), 86–93. 10.1016/j.psicoe.2017.12.001

[bjep12604-bib-0092] Zych, I. , Ortega‐Ruiz, R. , & Del Rey, R. (2015). Systematic review of theoretical studies on bullying and cyberbullying: Facts, knowledge, prevention, and intervention. Aggression and Violent Behavior, 23, 1–21. 10.1016/j.avb.2015.10.001

