# Supplementary material for: What roles matter? An explorative study on bullying and cyberbullying by using the eye‐tracker
Source: Br J Educ Psychol. 2023 Apr 26;95(2):249–69. doi: 10.1111/bjep.12604 (PMC12068039; doi:10.1111/bjep.12604)
Supplement: Supplementary file 1 — Data S1: [file BJEP-95-249-s001.docx]

Supplementary Material


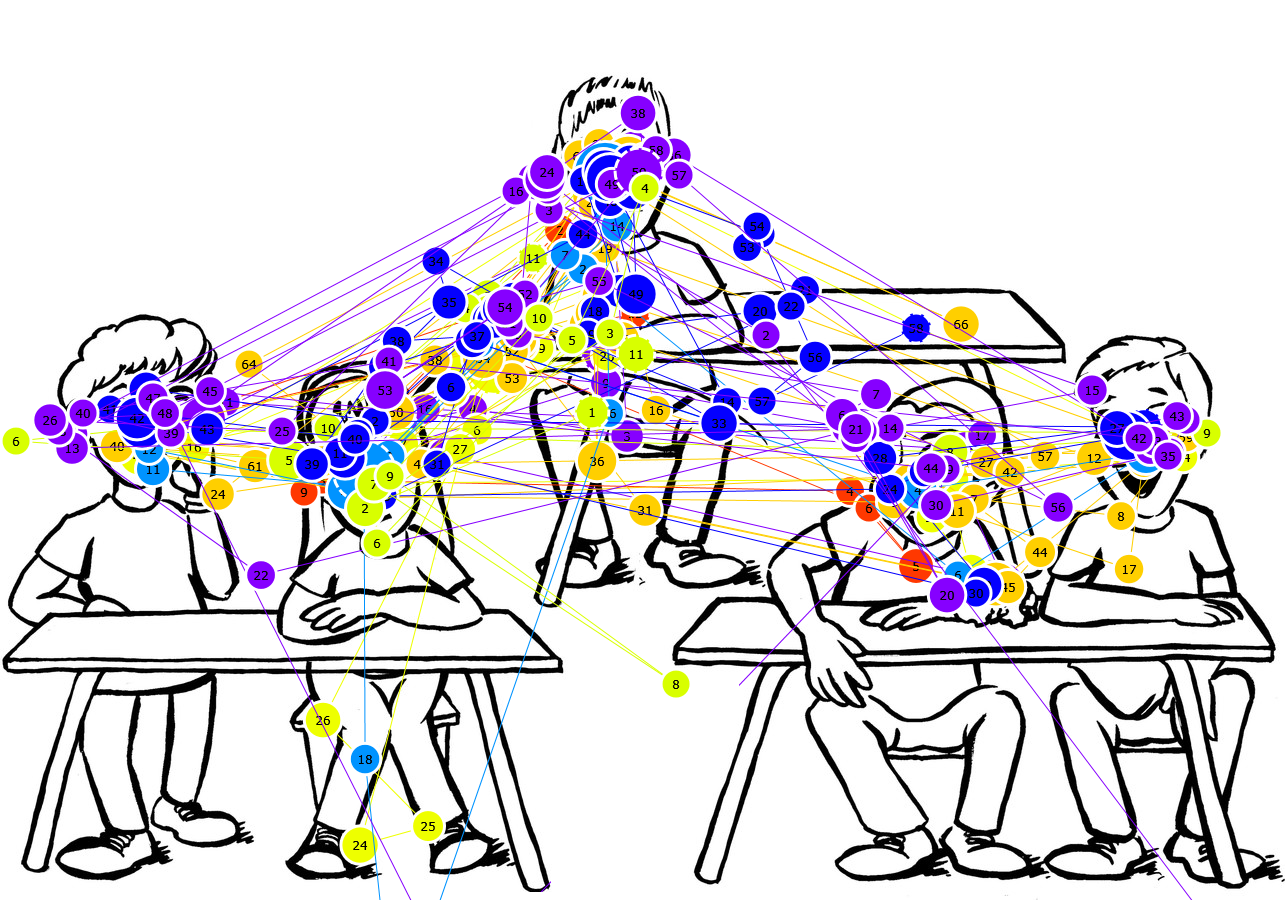


The picture shows the graphical representation of the eye movements of 10 students as they observed a vignette of physical bullying. The decision not to include the eye movements of all participants was made to avoid excessive overlap and maintain the clarity of the vignette.
